# Significance of experts' overall ratings for medical student competence in relation to history-taking

**DOI:** 10.1590/S1516-31802006000200010

**Published:** 2006-03-02

**Authors:** Luiz Ernesto de Almeida Troncon

**Keywords:** Clinical competence, Medical history taking, Educational measurement, Medical students, Medical education, Competência clínica, Anamnese, Avaliação educacional, Estudantes de Medicina, Educação médica

## Abstract

**CONTEXT AND OBJECTIVE:**

Overall ratings (ORs) of competence, given by expert physicians, are increasingly used in clinical skills assessments. Nevertheless, the influence of specific components of competence on ORs is incompletely understood. The aim here was to investigate whether ORs for medical student history-taking competence are influenced by performance relating to communication skills, completeness of questioning and asking content- driven key questions.

**DESIGN AND SETTING:**

Descriptive, quantitative study at Faculdade de Medicina de Ribeirão Preto, Universidade de São Paulo.

**METHODS:**

Thirty-six medical students were examined in a 15-station high-stake objective structured clinical examination (OSCE). At four stations devoted to history-taking, examiners filled out checklists covering the components investigated and independently rated students' overall performance using a five-point scale from 1 (poor) to 5 (excellent). Physician ratings were aggregated for each student. Nonparametric correlations were made between ORs.

**RESULTS:**

ORs presented significant correlations with checklist scores (Spearman's rs = 0.38; p = 0.02) and OSCE general results (rs = 0.52; p < 0.001). Scores for "communication skills" tended to correlate with ORs (rs = 0.31), but without reaching significance (p = 0.06). Neither the scores for "completeness" (rs = 0.26; p = 0.11) nor those for "asking key questions" (rs = 0.07; p = 0.60) correlated with ORs.

**CONCLUSIONS:**

Experts' overall ratings for medical student competence regarding history-taking is likely to encompass a particular dimension, since ratings were only weakly influenced by specific components of performance.

## RESUMO

**CONTEXTO E OBJETIVO:**

A avaliação global (AG) da competência de examinandos, feita por especialistas, tem sido utilizada em exames de habilidades clínicas, mas o significado desta medida é incerto. Neste trabalho foram investigadas as relações entre a AG de estudantes de Medicina na tomada da história clínica e medidas específicas do desempenho em três habilidades: comunicação, interrogatório completo e elaboração de perguntas essenciais, ligadas ao problema clínico.

**TIPO DE ESTUDO E LOCAL:**

Estudo descritivo quantitativo realizado na Faculdade de Medicina de Ribeirão Preto, Universidade de São Paulo.

**MÉTODOS:**

Trinta e seis estudantes de Medicina foram submetidos a exame clínico objetivo estruturado ("OSCE") em 15 estações, como avaliação prática final em disciplina compulsória. Em quatro estações de avaliação da obtenção da história clínica, os examinadores preencheram protocolo de observação referente às três habilidades investigadas e também atribuíram nota de AG com escala de cinco pontos (de 1 = "muito ruim" a 5 = "excelente"). Foram feitas correlações entre a AG e os escores para cada habilidade investigada, expressas pelo coeficiente de Spearman (rs).

**RESULTADOS:**

Houve tendência a correlação entre a AG e os escores para habilidades de comunicação (rs = 0,31; p = 0,06). Não houve correlação entre a AG e os escores para interrogatório completo (rs = 0,26; p = 0,11) e para a habilidade de fazer perguntas essenciais (rs = 0,07; p = 0,60).

**CONCLUSÕES:**

A avaliação global da competência de estudantes de Medicina na obtenção da história clínica, feita por professores de Medicina, constitui, provavelmente, uma dimensão específica, uma vez que parece ser pouco influenciada por componentes individuais do desempenho.

## INTRODUCTION

Assessment of clinical skills has a central role in both undergraduate and postgraduate medical education, as well as in professional certification. Objective methods for assessing clinical skills performance, such as the Objective Structured Clinical Examination (OSCE)^1^ or the Clinical Skills Assessment^2^ are widely used for evaluating the competence of students^3^ and residents^4^, as well as for qualifying medical graduates.^5^ In a typical objective examination of clinical skills, examinees rotate through a number of stations staffed by either real or standardized patients^6^, where they are required to perform different clinical tasks either in a focused or in a more comprehensive fashion. The examinees are observed and their performance is assessed using structured checklists covering specific components of performance.^1^ More recently, overall assessment of general performance, expressed as ratings given by expert physicians,^7^ standardized patients^8^ or even real patients^9^ and appended to station checklists, has been shown to have a better construct validity than checklists, while maintaining satisfactory estimates of reliability.^10^

While a number of studies have explored the quality of ratings given by expert physicians,^7,11^ expressed in terms of validity and reliability, the influence of specific components of competence upon this overall rating is incompletely understood. The present study therefore investigated whether overall ratings given by expert physicians for student history-taking competence are influenced by performance in relation to three specific components: a) communication skills; b) completeness of questioning; and c) asking content-driven key questions.

## METHODS

### Settings

The current local medical curriculum in our medical school comprises two years of integrated basic sciences, one semester (in the third year) of preclinical disciplines and three semesters (in the third and fourth years) of clinical disciplines, before the internships run during the two final years (fifth and sixth years). The clinical disciplines integrate medical and surgical subjects into larger fields, such as Cardiovascular Diseases or Respiratory Disorders, and are developed mainly through practical activities in wards and outpatient clinics. In the clinical discipline relating to Digestive Diseases, student assessment is carried out in accordance with international recommendations^12^ and using an OSCE model that was introduced into this medical school nearly 10 years ago.^13^

### Study design

The data for this study came from a high-stake OSCE used as the final examination for the clinical discipline of Digestive Diseases (fourth year students). Students need to pass this examination in order to be eligible to start the internship period. The data utilized was from a group of 36 medical students of both sexes, aged 21-25 years, who were assessed under the same conditions. This OSCE comprised 15 seven-minute stations, including four with simulated patients for the assessment of history-taking skills. Another four stations had real patients, with true signs, for the assessment of physical examination skills. The remaining stations utilized clinical vignettes and photographs or radiographs for assessing both pattern recognition and clinical reasoning. In all stations, a small set of questions was used to assess students' abilities to detect relevant findings from the presentation of the patient or illustration, and to reason on the data obtained.

In the four stations designed to assess history-taking skills, experienced physicians observed and evaluated student performance using predetermined detailed checklists containing 10 to 14 items. These checklists contained four standard items relating to communication and interaction with the patient and a number of different items covering the relevant subjects that were expected to be addressed in the interview, according to the specific station content. For the four stations designed for this examination, the tasks and contents were as follows: a) to characterize symptoms in an adult male patient presenting with heartburn, regurgitation and dyspepsia; b) to characterize symptoms in an adult female with acute diarrhea; c) to explore risk factors related to habits and lifestyle for a male adult with recently diagnosed chronic hepatitis B virus; and d) to characterize bowel habits and stool features of a child with chronic, persistent diarrhea through interviewing the mother.

Four standardized patients who had been appropriately trained according to accepted recommendations^14^ staffed these four stations devoted to assessing history-taking skills. All the standardized patients had already portrayed cases in previous examinations. In each station, one experienced physician worked as the student examiner. There were two professors of medicine, one assistant professor of gastroenterology and one associate professor of pediatrics. The examiner at each station filled out checklists covering the components investigated, and also independently rated the student's overall clinical performance using a five-point scale, from 1 (poor) to 5 (excellent). This rating was appended at the bottom of the checklist. The examiners were unaware of the aims of this investigation. The influence of specific components of performance on the overall ratings was determined by calculating the correlations between the relevant checklist data and the overall rating scores.

### Data analysis

The results from the checklists and overall ratings were analyzed independently. From the checklists, the following scores were obtained: a) overall performance, represented by the sum of all items; b) performance in communication skills, represented by the four standardized items specifically designed with this aim; c) completeness of questioning, represented by the score for overall performance minus the score for communication; d) performance in asking key questions, represented by two to four items that were identified in each station as being highly relevant to that particular clinical context. Overall ratings given by the expert physician at each station were averaged to form a single aggregated score for each student. An overall OSCE performance score was obtained by averaging the results from all 15 stations, after recalculation by subtracting the overall rating component for these four stations. All data were normalized and converted to percentages.

### Statistical analysis

Since data for some variables did not pass the Kolmogorov-Smirnov normality test, the results were analyzed using non-parametric methods. The Kruskal-Wallis test was utilized for analyzing the differences between the experts' overall ratings, with subsequent application of Dunn's multiple comparisons test. Correlations between overall ratings and either checklist data or overall OSCE results were estimated by means of Spearman's coefficient. All calculations were carried out using dedicated software (Graph Pad Instat, Prism, United States). Differences were taken to be statistically significant when the p- values were less than or equal to 0.05.

## RESULTS

All the students passed the whole examination. At each of the four history-taking stations, no more than three different students obtained an unsatisfactory score for that station. Only one student obtained an unsatisfactory score for more than one of these stations.

The overall ratings given by the four expert physicians to students are shown in Table 1. Analysis of variance showed a significant difference between examiners (p = 0.03), with one of them (number 2) giving significantly higher (p < 0.05) ratings than the others.

**Table 1:** Overall ratings for history-taking skills given by four expert physicians to 36 medical students assessed in four different seven-minute stations. Results are expressed as percentages

Examiner	Mean (SD)	Median (Range)
1	60 (17.3)	75 (25 – 75)
2	77 (17.4)*	75 (25 – 100)*
3	61 (21.0)	50 (25 – 100)
4	59 (16.1)	50 (25 – 75)
**Aggregated scores**	**64 (6.2)**	**62 (50 – 81)**

*SD = standard deviation*.

* *Significantly different from other examiners (p = 0.05, Kruskal-Wallis and Dunn's tests)*;

The data extracted from the different components of the checklists are shown in Table 2, which also contains the overall OSCE result. Although there was a trend towards improved student performance regarding asking "key questions", the differences between the three components were not statistically significant.

**Table 2:** Results for 36 medical students taking a comprehensive objective structured clinical examination (OSCE) for clinical skills in digestive diseases. The data presented for communication skills, completeness of questioning, asking key questions and overall checklist represent aggregated results obtained from four different seven-minute stations designed for assessing history-taking skills. Results are expressed as percentages

Data	Mean (SD)	Median (Range)
Communication	75 (7.2)	75 (61 – 86)
Completeness of questioning	74 (6.1)	75 (57 – 86)
Asking key questions	85 (4.7)	87 (75 –100)
Overall checklist	73 (4.5)	74 (61 - 81)
Overall OSCE results	72 (7.1)	73 (56 – 86)

*SD = standard deviation.*

The values for the various correlation coefficients calculated are shown in Table 3. The aggregated overall ratings presented positive, statistically significant correlations with the data from the whole checklist and the overall OSCE results. Scores for "communication skills" extracted from checklists tended to correlate with aggregated overall ratings (rs = 0.31), but without reaching significance (p = 0.06). Neither the checklist scores for "completeness of questioning" nor those for "key questions" correlated significantly with aggregated overall ratings.

**Table 3:** Spearman's coefficient of correlation (r) between overall ratings for history-taking skills and checklist scores for specific components of performance. The overall ratings were given by four expert physicians to 36 medical students taking a comprehensive OSCE for clinical skills in digestive diseases. Correlations between overall ratings and overall checklist and OSCE results are also presented

Data	r (95% CI)	p
Communication	0.31 (- 0.02 – 0.58)	0.06
Completeness of questioning	0.26 (- 0.08 – 0.55)	0.11
Asking key questions	0.07 (- 0.27 – 0.39)	0.68
Overall checklist	0.40 (0.09 – 0.64)	0.01*
Overall OSCE results	0.51 (0.21 - 0.73)	0.001*

* *Significantly different from zero*;

*CI = confidence interval*.

## DISCUSSION

Overall ratings given by expert physicians, which are extensively used in in-training assessment of interns and residents,^15^ were introduced into objective, structured examinations of clinical competence as a way of capturing a more comprehensive and relevant dimension of student or graduate performance, in addition to checklist data.^9,10^ A number of studies have demonstrated that overall ratings are valid and reliable, and also that they seem indeed to be particularly suited to recording both examinees' attitudes towards patients and their approaches to given clinical problems.^7-10^ Studying what determines the experts' overall ratings is important not only for obtaining better quality information regarding assessment, but also for improved focus in the feedback to examinees. This has increasingly been incorporated into objective examina- tions,^16^ thus increasing the educational value of assessment procedures.^3^

The present study investigated whether overall ratings attributed by expert physicians to medical students' history-taking skills were influenced by specific components of performance. The examiners, as experienced physicians, were familiar with the structured clinical situations included in objective examinations, and were regarded as capable of making a proficient holistic judgment about how appropriate the examinee's approach to the patient and the clinical problem was.^11^ The standardized patients staffing the various stations of the examination were also familiar with their roles, since they had often served in previous examinations.

The present study found that neither communication skills, nor the completeness of questioning or asking of essential content-driven questions correlated significantly with overall ratings. The performance measured by checklist items covering the ability to ask essential questions, defined by clinical context, showed virtually no correlation with overall ratings. On the other hand, communication skills showed the highest positive correlation value with overall ratings. This might suggest that overall ratings are more affected by interpersonal skills, rather than technical characteristics. Nevertheless, statistical significance for the correlation between overall ratings and communication skills was not reached, which means that no conclusion can yet be reached regarding this matter.

The use of analytical overall ratings with different component subscales, as proposed recently^17^ would make the different determinants of experts' overall ratings clearer. Nevertheless, this would most likely deprive overall ratings of their holistic meaning, and would also be technically more difficult to reconcile with checklist recordings during the examination.

The finding in the present study of significant positive correlations between the experts' overall ratings and both the checklist scores and the overall OSCE results is in agreement with data from several other studies.^9,10,18^ This indicates that overall ratings are valid measurements of clinical competence regarding history-taking. As far as reliability is concerned, the relatively small number of stations and examiners in the present study precluded the use of more accurate estimation methods such as Cronbach's internal consistency and generalizability coefficients.^19^ Nevertheless, the overall ratings given by three out of the four examiners were similar and the averaging of the individual ratings probably minimized any inferred influence from discordance on the present results.

On the other hand, some limitations of the present study should be noted. In addition to the relatively small number of stations and examiners already mentioned, the examination covered only material relating to digestive diseases. It is well known that the practical performance relating to the approach adopted towards patients is dependent on the content of the clinical problem involved.^20^ Also, a relatively high degree of general clinical competence at the expected level was observed among the students in the present study, which was expressed by the unusually low failure rate. It would be thus interesting to confirm these findings in examinations that included a broader range of material and greater diversity of clinical competence level among the students.

## CONCLUSIONS

The data from the present study did not show any significant correlation between the performance components investigated and the experts' overall ratings for student competence in history-taking. This suggests that this holistic measurement encompasses a particular dimension that deserves further investigation.
